# Integration of genome-wide mRNA and miRNA expression, and DNA methylation data of three cell lines exposed to ten carbon nanomaterials

**DOI:** 10.1016/j.dib.2018.05.107

**Published:** 2018-05-25

**Authors:** Giovanni Scala, Veer Marwah, Pia Kinaret, Jukka Sund, Vittorio Fortino, Dario Greco

**Affiliations:** a Faculty of Medicine and Life Sciences, University of Tampere, Finland; b Institute of Biosciences and Medical Technology (BioMediTech), University of Tampere, Finland; c Institute of Biotechnology, University of Helsinki, Finland; d Institute of Biomedicine, University of Eastern Finland, Kuopio Campus, Finland

## Abstract

We present data derived from an exposure experiment in which three cell-lines representative of cell types of the respiratory tissue (epithelial type-I A549, epithelial type-II BEAS-2B, and macrophage THP-1) have been exposed to ten different carbon-based nanomaterials for 48 h.

In particular, we provide: genome-wide mRNA and miRNA expression, and DNA methylation; gene tables, containing information on the aberrations induced in these three genomic data layers at the gene level; mechanism of action (MOA) maps representing the comparative functional alteration induced in each cell line and each exposure.

**Specifications Table**

**Table:** 

Subject area	*Biology*
More specific subject area	*Nanotoxicology*
Type of data	*Tables, figures, omics data matrices*
How data was acquired	*Microarray based assays*
Data format	*Raw, analyzed*
Experimental factors	*Cells were cultured with corresponding media and supplements. THP-1 cells were differentiated with* 50 nM *PMA for* 48 h *before treatments.*
Experimental features	*Cells were exposed to* 10 µg/ml *of* 10 *different carbon nanomaterials for* 48 h*. DNA, mRNA and miRNA were extracted, purified and quality checked for arrays.*
Data source location	*University of Tampere, Finland*
Data accessibility	*Figures and tables are in this article. Raw and processed microarray data are available through Array Express repository (Accession Numbers ArrayExpress: E-MTAB-6396, E-MTAB-6406, E-MTAB-6397)*

**Value of the data**

• Omics datasets can be used to integrate and compare molecular alterations consequent to nanomaterials exposure studies.
• Gene (expression) tables can serve as a reference in future studies modelling the cell specific response of each gene at different molecular layers.
• MOA maps can be used as a starting point to draft adverse outcome pathways (AOP) that take into account cell type-specific responses.

## 1 Data

The data presented in this paper includes three sets of microarray data for 33 mRNA, miRNA and methylation samples available on ArrayExpress platform, 30 gene tables containing multi-omic differential information on these three layers for human genes, and 3 maps representing functional alteration of all exposures at the pathways level.

Microarray data samples summarized in Table 1 are composed of 96 raw and preprocessed data matrices reporting mRNA expression values for refseq genes in control and exposed cell lines, 91 raw and preprocessed matrices reporting mirRNA expression values in control and exposed cell lines and 99 raw and preprocessed matrices reporting DNA methylation values at the CpGs level in control and exposed cell lines.

**Table 1:** Summary of array data.

Accession	Type	# samples	Platform	RAW data	Preprocessed data
E-MTAB-6396	mRNA	96	Agilent SurePrint G3Human GE 8×60K	Yes	Yes
E-MTAB-6406	miRNA	91	Agilent SurePrint G3 Unrestricted Human miRNA_V21 8×60K	Yes	Yes
E-MTAB-6397	DNA methylation	99	Illumina HumanMethylation450 BeadChip	Yes	Yes

For each data layer and each exposure, we performed a differential analysis between the control and the exposed samples with limma linear models and annotated a list of 22,789 human gene symbols where we summarized DNA methylation change (p-value and log fold-change) in promoter and body regions; targeting miRNAs expression changes (p-value and log fold-change), symbolically linked to the gene body region; and mRNA expression changes (p-value and log fold-change). All these values were used to compute a cumulative score for the gene determining the overall impact of all molecular aberrations on any given particular gene. Supplementary Table S1 is an excel file reporting the summary information shown in Table 2 in the first sheet, as well as the above described annotation for each exposure in the remaining 30 sheets.

**Table 2:** Multi-omic gene annotation data.

Field	Explanation
test_annotation.score_data.genes	Gene symbol
scores	SMITE score
methylation_promoter_effect	Combined methylation log fold-change for promoter region
methylation_body_effect	Combined methylation log fold-change for body region
mirna_body_effect	Combined log fold-change of targeting miRNAs
expression_effect	MRNA expression log fold-change
methylation_promoter_pvalue	Combined methylation p-value for promoter region
methylation_body_pvalue	Combined p-values of targeting miRNAs
mirna_body_pvalue	Combined methylation p-value for body region
expression_pvalue	MRNA expression p-value

The functional alteration map data (Fig. S1–S3) report, for each exposure, the KEGG pathways significantly enriched from the high scoring genes. Each pathway is annotated with the leading direction (red for upregulation and green for downregulation) of expression change of its genes for the corresponding exposure. Pathways are grouped based on KEGG hierarchical structure in six categories: “Metabolism”, “Genetic Information Processing”, “Environmental Information Processing”, “Cellular Processes”, “Organismal Systems” and “Human Diseases”. Fig. S1 reports the pathways enriched from all altered genes. Fig. S2 and Fig. S3 report pathways enriched in the two partitions of genes (concordant and discordant), based the adherence with a set of general rules of interaction linking the induced changes in DNA methylation levels and miRNA expression levels with observed changes in gene expression levels. In particular, we defined a gene alteration to be “concordant” if its expression upregulation was coupled with hypomethylation in the promoter region, hypermethylation in the gene body region, or with downregulation of a microRNA specifically predicted to potentially target that gene. Likewise, we defined a gene alteration to be “concordant” if its expression downregulation was coupled with hypermethylation in the promoter region, or with upregulation of a microRNA specifically predicted to potentially target that gene, regardless of the methylation status of the gene body. The genes not following these rules were classified to be “discordant”, and their alteration was hypothesized to be not under the control of DNA methylation or microRNA expression, but other unknown regulatory factors, such as histone modifications.

## 2 Experimental design, materials and methods

### 2.1 Cell cultivation

THP-1 cells (ATCC TIB-202) were cultivated in complete RPMI 1640 media (Gibco, Thermo Fisher Scientific, Life Technologies, USA) supplemented with 10% FBS (Gibco, USA) and 1% Ultraglutamine (Gibco, USA) and differentiated with 50 nM PMA (phorbol-12-myristate-13-acetate) for 48 h before exposures. BEAS-2B (American Type Culture Collection through LGC Promochem AB (Borås, Sweden)) were cultured in LHC-9 media (Gibco, USA) and A549 cells (ATCC CCL-185) were grown in DMEM media (including L-glutamine, Gibco, USA) supplemented with 10% FBS (Gibco, USA).

### 2.2 Exposure settings

Exposures were performed on 12-well plates, with 10 µg/ml nanomaterial concentration for 48 h.

**THP-1**: 800,000 cells per well for RNA and DNA and 900,000 cells/well to miRNA extractions; **BEAS-2B**: 100,000 cells/well to RNA, DNA and miRNA extractions; **A549**: 50,000 cells/well to RNA, DNA and miRNA extractions.

### 2.3 RNA and DNA extraction protocols

After 48 h exposure, cells were washed with PBS, and lysed (Qiagen lysing buffer). DNA, RNA and miRNA were extracted using Qiagen extraction kits: Qiagen AllPrep 96 DNA/RNA extraction kit for mRNA and DNA and Qiagen miRNeasy 96 extraction kit for miRNA (Qiagen, Germany). Quality of the RNA was confirmed by NanoDrop (ND-1000, Thermo Fisher Scientific Inc., Wilmington, NC, USA) and Bioanalyzer (Agilent Technologies, USA). RNA samples with high RNA integrity values (> 9) were used in microarray analyses.

### 2.4 Experimental settings

Low Input Quick Amp, two-color microarray-based gene expression protocol: 100 ng of total RNA labeled with Cy3 or Cy5 dyes were hybridized to Agilent SurePrint G3Human GE 8×60K DNA microarrays (Agilent, USA).

miRNA Microarray System with miRNA Complete Labeling and Hyb Kit protocol: 100 ng of miRNA labeled with Cyanine 3-pCp dye and hybridized to Agilent SurePrint G3 Unrestricted Human miRNA_V21 8×60K microarrays (Agilent, USA).

Methylation protocol: 500 ng of DNA was bisulfite converted with the EZ-96 Methylation Kit Bisulfite-treated DNA was amplified, fragmented and hybridized to the HumanMethylation450 BeadChip (Illumina, USA).

## 3 Microarray data

**Fig. 1:**
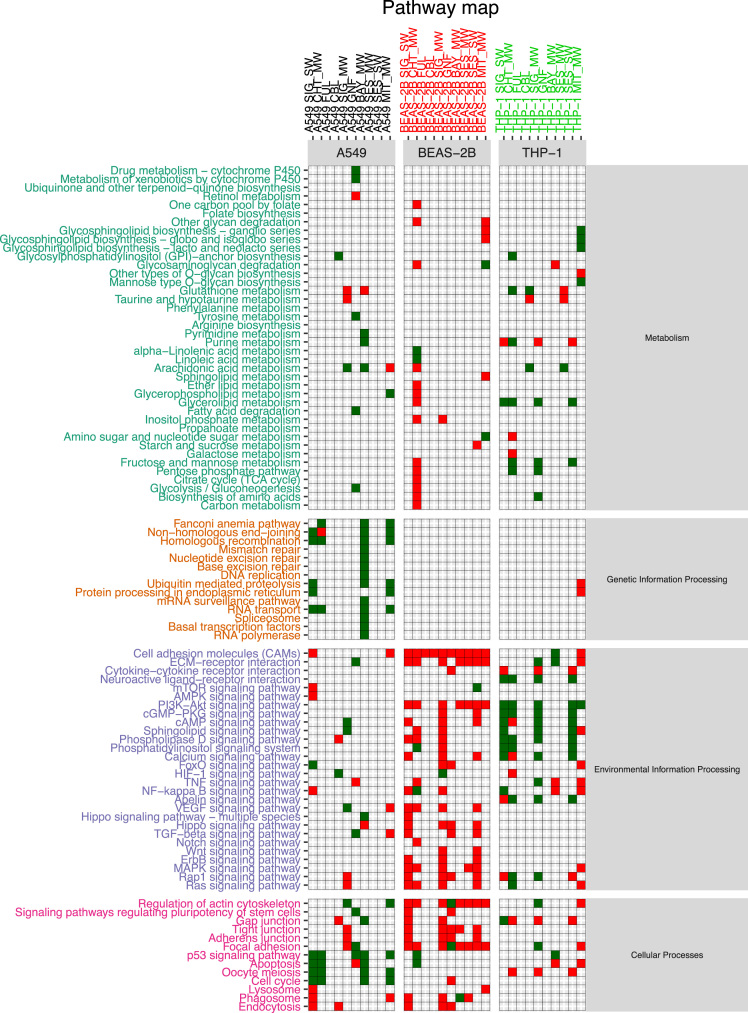

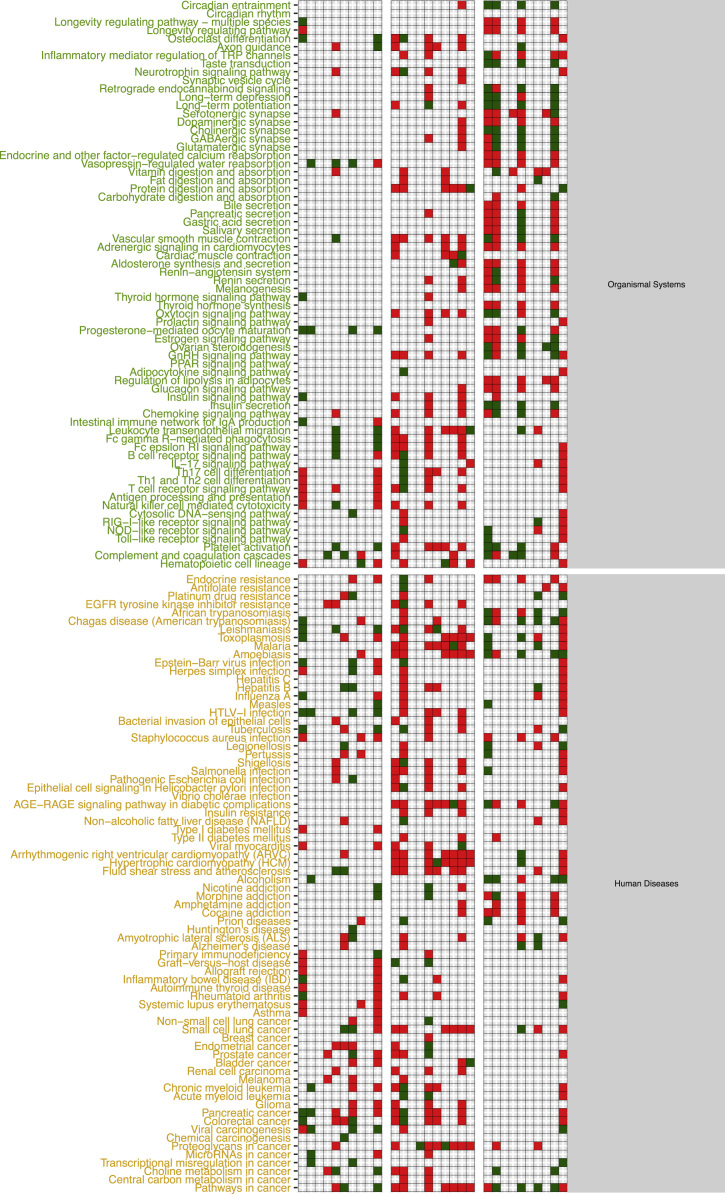

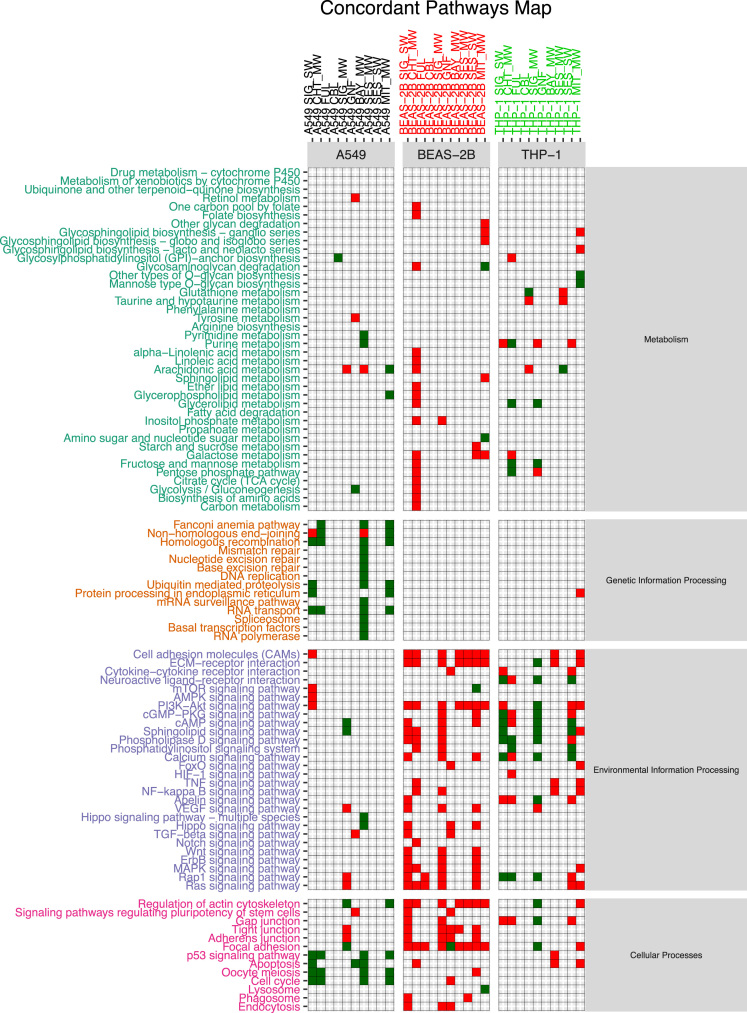

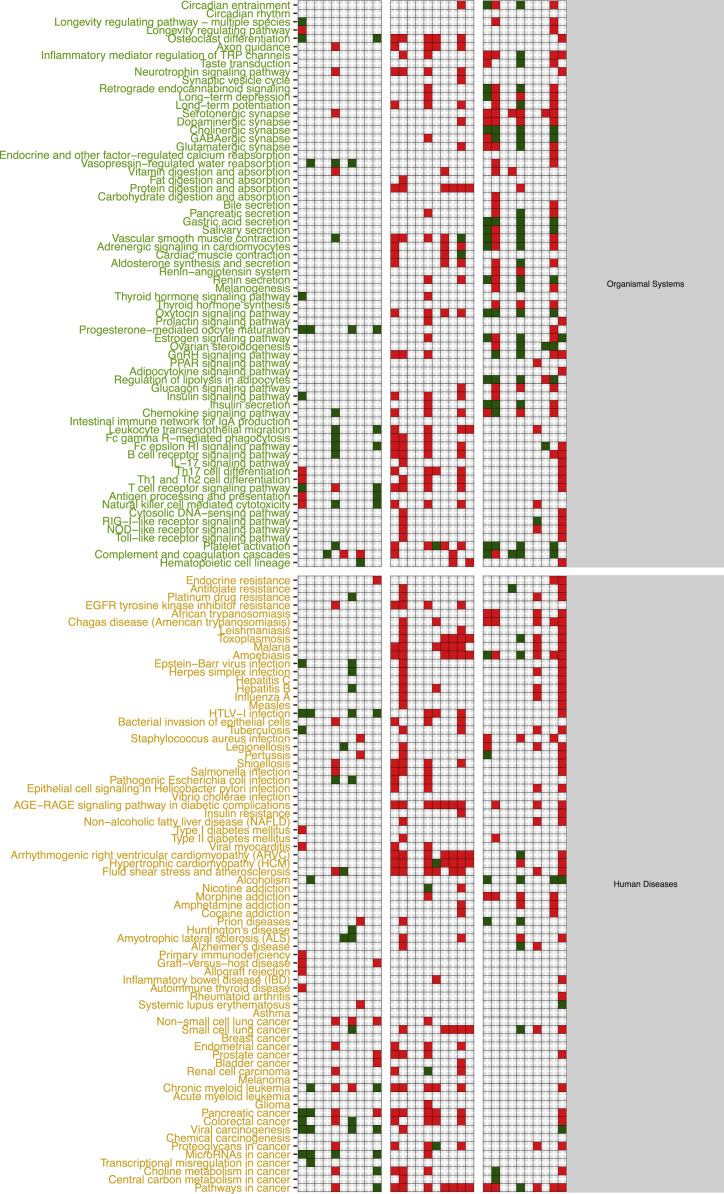

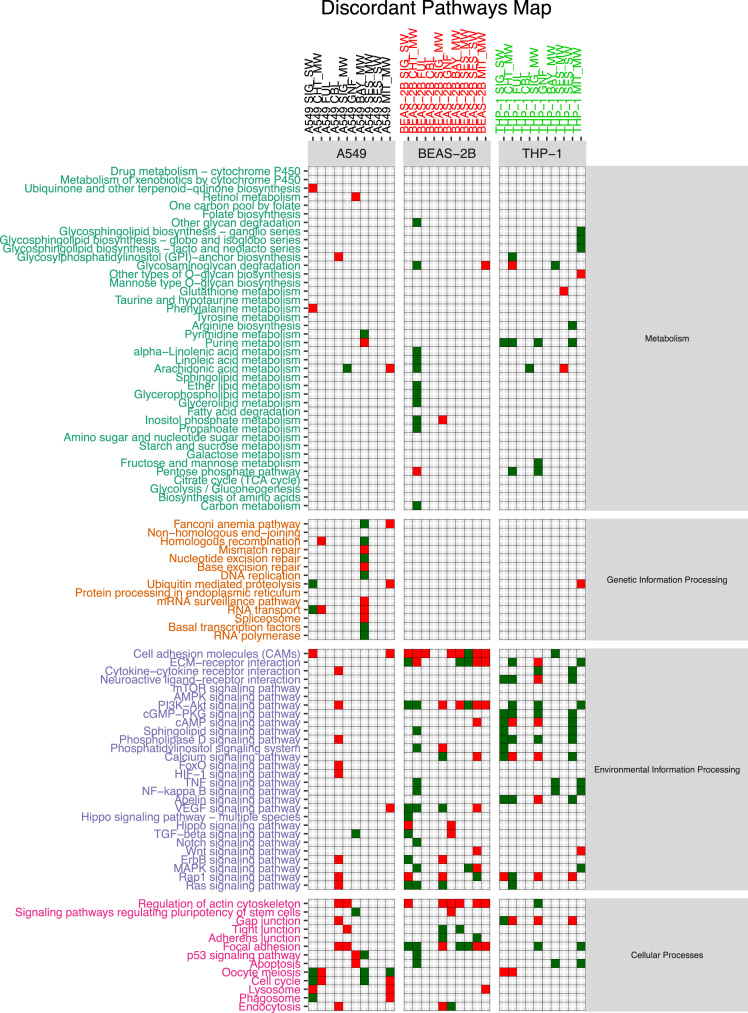

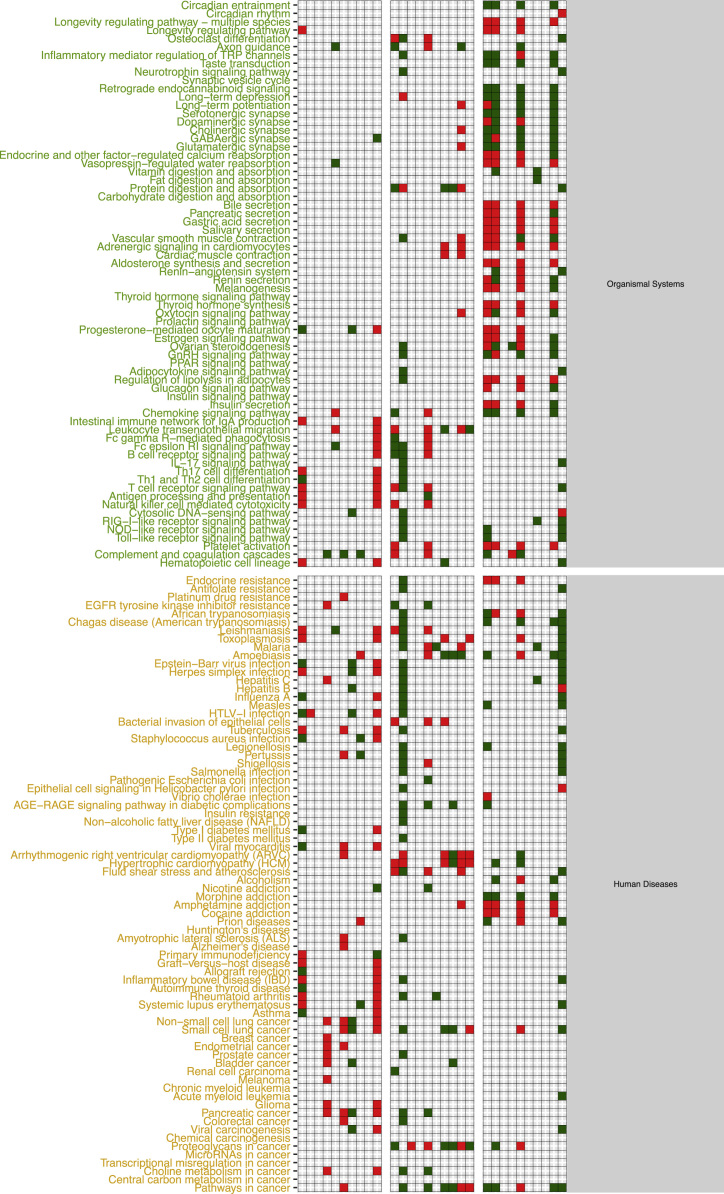
Data generation scheme**.** Workflow of data generation: microarray preprocessing and analysis of single layers is reported in panel A; data integration and generation of scored gene tables is reported in panel B; gene module detection and functional profiling of each exposure is reported in panel C.

Microarray data (Fig. 1, panel A), has been imported, preprocessed and analyzed using R as follows.

mRNA raw data has been imported using limma read.maimages, quality filtered based on negative probes distribution values, quantile normalized, log2 transformed and median aggregated at RefSeq gene level using the corresponding Agilent annotation file. Batch effect removal of known technical batch effects been performed by using Combat method from the SVA package [1].

miRNA raw data has been quality filtered based on negative probes distribution values, quantile normalized, log2 transformed and median aggregated at miRbase miRNA ids level. Batch effect removal of known technical batch effects been performed by using Combat method from the SVA package. [1] Differential expression analysis between each exposure and the corresponding controls has finally been performed using a limma model from limma package.

Methylation data has been preprocessed with minfi package. [2] Briefly, raw data has been imported from idat files, probes were filtered by keeping those having a detection p-value less than 0.01 in all samples. Data was then normalized using SWAN method [3], converted to M values and filtered for probes having a SNP in the interrogation or the extension site and probes known to be prone to as cross-hybridization problems [4].

Batch effect removal was performed by using sva function from SVA package [1] to detect the presence of surrogate variables, the obtained surrogate variables value has then been discretized into n_samples^(1/3) bins by using the discretize function from infotheo package [5] and finally corrected using ComBat method from the SVA package [1].

## 4 Multi-omic gene annotation data

For each of the three analyzed layers, differential expression (DNA methylation) analysis between each exposure and the corresponding controls has been performed using a limma model from limma package. [4].

Data in Supplementary Table S1 (Fig. 1, panel B) has been obtained by integrating differential expression and methylation results using custom scripts and SMITE package. [6] In particular, differential expression and methylation data (in the form of p-values and log fold changes) has been annotated to each UCSC gene transcription start site [ TSS − 1 kb, TSS + 1 kb] and gene body region [TSS + 1 kb, TES] as follows.

CpG methylation has been associated in SMITE to TSS and body regions of genes by using their genomic location, while miRNAs have been symbolically associated with the gene bodies of their top 10% target genes using t-scores form TargetScan database [7].

A score has finally been assigned to each gene by integrating the expression p-value and fold-change with the same values from the two modification layers using in SMITE the weights shown in Table 3.

**Table 3:** SMITE weights.

Feature/relationship	mRNA Expression	Gene promoter methylation	Gene body methylation	Targeting miRNA expression
Relationship with mRNA level	Direct correlation	Inverse correlation	Direct correlation	Inverse correlation
Weight	0.70	0.15	0.05	0.10

## 5 MOA maps data

Data presented in Figs. S1–S3 (Fig. 1, panel C) has been obtained by using scored gene lists from Supplementary Table S1. In particular given a scored list of gene for a particular exposure comparison, we used SMITE to detect modules of high scoring genes using a SpinGlass algorithm with 1000 randomizations on the Reactome52 interaction network [8]. We then derived, for each exposure, the KEGG pathways enriched for the all the genes detected from the obtained set of modules. Given an exposure, each enriched pathway was classified as up- or down-regulated if the sign of the median mRNA expression change of the genes in the pathway was respectively positive or negative.

Fig. S1 report the map of all enriched pathways, divided by exposure and cell line, obtained by taking in consideration the whole set of genes from the corresponding table when computing the modules.

Figs. S2 and S3 were generated by only using the modules computed from a subset of the genes from each exposure, respectively the concordant and the discordant genes.

Given an exposure and its associated table in Supplementary Table S1, concordant genes were defined as up-regulated genes with hypo-methylated promoter or targeted by down-regulated miRNAs or down-regulated genes with hyper-methylated promoter or up-regulated targeting miRNAs. Discordant genes are defined as the complement of the concordant gene set with respect to the starting table.

All the data and associated statistics presented in this manuscript has been produced using R software environment. [9] The R code used to generate these datasets is provided in Supplementary file S2.

## Appendix A Supplementary material

Figure S1. Enriched pathways resulting from each exposure grouped by pathway category on rows and by exposed cell line on columns. Red and green cells are associated with significantly enriched KEGG pathways (FDR adjusted hypergeometric *p*-value < 0.05), grey cells stand for no significant enrichment. Red cells are associated with enriched KEGG pathways whose genes have a positive median log fold-change in the corresponding comparison, green cells are associated with KEGG pathways whose genes have a negative median log fold-change in the corresponding comparison.

.

Figure S2. Enriched pathways resulting from each exposure using only concordant genes grouped by pathway category on rows and by exposed cell line on columns. Red and green cells are associated with significantly enriched KEGG pathways (FDR adjusted hypergeometric *p*-value < 0.05), grey cells stand for no significant enrichment. Red cells are associated with enriched KEGG pathways whose genes have a positive median log fold-change in the corresponding comparison, green cells are associated with KEGG pathways whose genes have a negative median log fold-change in the corresponding comparison.

.

Figure S3. Enriched pathways resulting from each exposure using only discordant genes grouped by pathway category on rows and by exposed cell line on columns. Red and green cells are associated with significantly enriched KEGG pathways (FDR adjusted hypergeometric *p*-value < 0.05), grey cells stand for no significant enrichment. Red cells are associated with enriched KEGG pathways whose genes have a positive median log fold-change in the corresponding comparison, green cells are associated with KEGG pathways whose genes have a negative median log fold-change in the corresponding comparison.

.

Table S1. Excel spreadsheet, reporting in each sheet differential analysis summaries and SMITE gene scores for each exposure. For each gene are reported in the order the gene symbol, the SMITE score, the combined methylation log fold-change for promoter region, the combined methylation log fold-change for body region, the combined log fold-change of targeting miRNAs, mRNA expression log fold-change, combined methylation p-value for promoter region, combined p-values of targeting miRNAs, combined methylation p-value for body region, mRNA expression p-value.

.

Supplementary file S2. Zip file containing all the scripts used to generate data in this manuscript.
