# Supplementary material for: Integration of genome-wide mRNA and miRNA expression, and DNA methylation data of three cell lines exposed to ten carbon nanomaterials
Source: Data Brief. 2018 May 25;19:1046–57. doi: 10.1016/j.dib.2018.05.107 (PMC6140287; doi:10.1016/j.dib.2018.05.107)
Supplement: Supplementary file 1 — Supplementary material [file mmc1.docx]

**Conflict of interest**

The authors have no competing interests to declare.
