# Supplementary figures and images for: Integration of genome-wide mRNA and miRNA expression, and DNA methylation data of three cell lines exposed to ten carbon nanomaterials
Source: Data Brief. 2018 May 25;19:1046–57. doi: 10.1016/j.dib.2018.05.107 (PMC6140287; doi:10.1016/j.dib.2018.05.107)

Pathway map

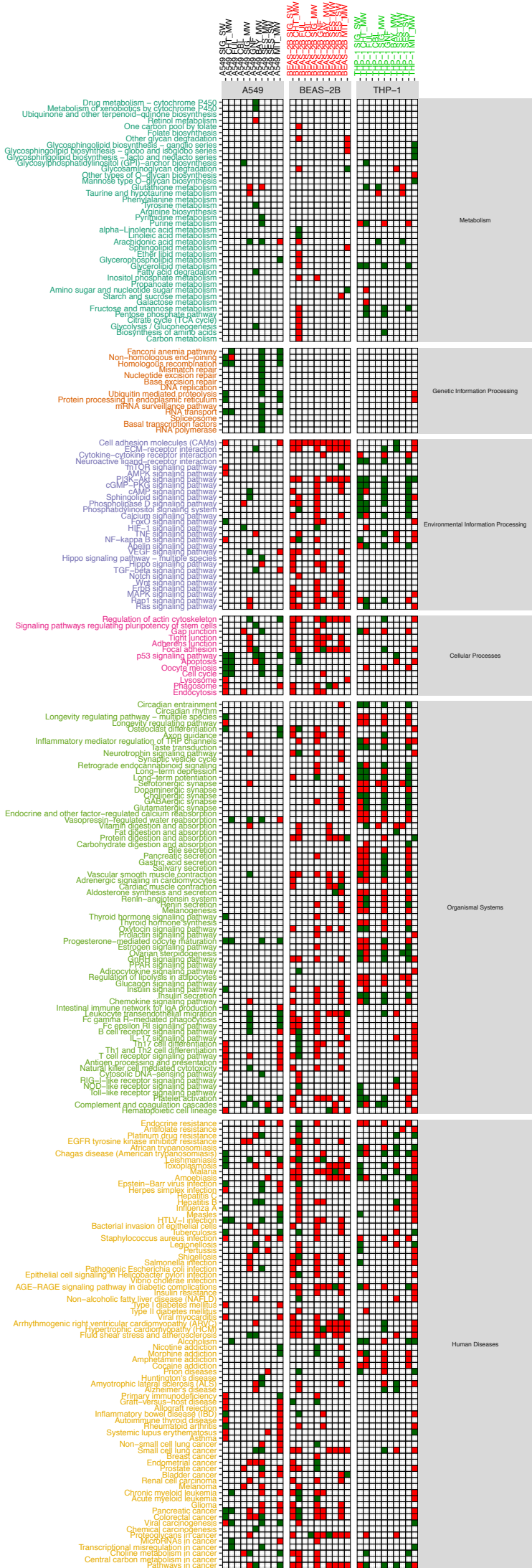

Supplement: Supplementary file 2 — Figure S1. Enriched pathways resulting from each exposure grouped by pathway category on rows and by exposed cell line on columns. Red and green cells are associated with significantly enriched KEGG pathways (FDR adjusted hypergeometric p-value < 0.05), grey cells stand for no significant enrichment. Red cells are associated with enriched KEGG pathways whose genes have a positive median log fold-change in the corresponding comparison, green cells are associated with KEGG pathways whose genes have a negative median log fold-change in the corresponding comparison. [file mmc2.pdf]

# Concordant Pathways Map

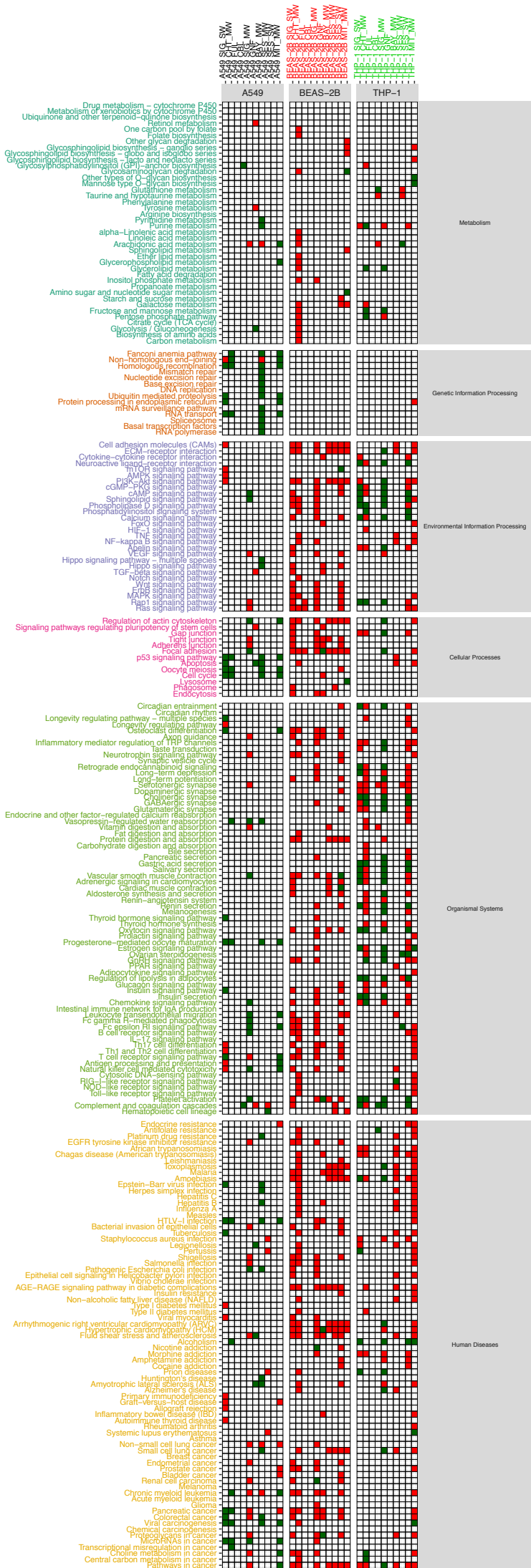

Supplement: Supplementary file 3 — Figure S2. Enriched pathways resulting from each exposure using only concordant genes grouped by pathway category on rows and by exposed cell line on columns. Red and green cells are associated with significantly enriched KEGG pathways (FDR adjusted hypergeometric p-value < 0.05), grey cells stand for no significant enrichment. Red cells are associated with enriched KEGG pathways whose genes have a positive median log fold-change in the corresponding comparison, green cells are associated with KEGG pathways whose genes have a negative median log fold-change in the corresponding comparison. [file mmc3.pdf]

Discordant Pathways Map

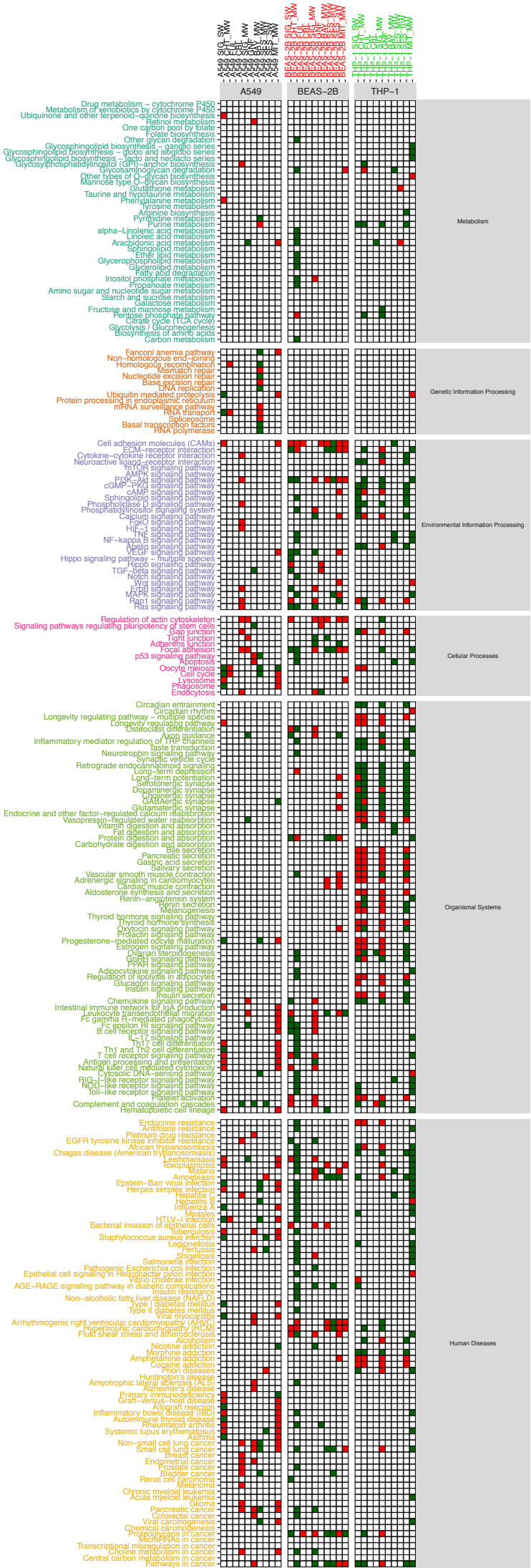

Supplement: Supplementary file 4 — Figure S3. Enriched pathways resulting from each exposure using only discordant genes grouped by pathway category on rows and by exposed cell line on columns. Red and green cells are associated with significantly enriched KEGG pathways (FDR adjusted hypergeometric p-value < 0.05), grey cells stand for no significant enrichment. Red cells are associated with enriched KEGG pathways whose genes have a positive median log fold-change in the corresponding comparison, green cells are associated with KEGG pathways whose genes have a negative median log fold-change in the corresponding comparison. [file mmc4.pdf]
